# Supplementary material for: Overcoming Barriers in the Introduction of Early Warning Scores for Prevention of In-Hospital Cardiac Arrests in Austrian Medical Centers
Source: Healthcare (Basel). 2025 Oct 18;13(20):2624. doi: 10.3390/healthcare13202624 (PMC12564850; doi:10.3390/healthcare13202624)
Supplement: Supplementary file 1 [file healthcare-13-02624-s001.zip › healthcare-3829844-supplementary.pdf]

Supplemental Materials

Interview guide

Design of an early warning system for in-hospital emergencies in adults in Austrian central and specialized hospitals

Priv.-Doz. Dr. Benedikt Treml

Concept
Topic and content of the expert interviews<br><br>In-hospital emergencies, especially cardiac arrest, are still associated with high mortality. A plethora of early warning systems seek for early detection of such critically ill patients. However, it is unclear to what extent such established and validated early warning systems can be transferred to the Austrian healthcare system. Furthermore, it is unclear whether they will be implemented across Austrian hospitals.<br>With the help of this guideline, experts in in-hospital emergency medicine in Austrian medical centers and specialized hospitals will be interviewed. The aim is to examine how early warning systems for in-hospital emergencies at Austrian medical centers and specialized hospitals can recognize emergencies at an early stage. Furthermore, it will be examined whether the use of the internationally established "MEWS" early warning system in the Austrian healthcare system is suitable to address local differences and make MEWS more effective.<br><br>Target group<br><br>Nurses and physicians who work as experts for in-hospital emergency medicine in an Austrian medical center or specialized hospital.

Interview guide	
**Beginning**	a) Outline of the topic<br>b) Duration and procedure of the interview<br>c) Data protection agreement for the protection of personal data
**Question 1**	How long have you been involved in in-hospital emergency medicine and what is your area of responsibility?
**Queries**	a) In which specialty do you work in your hospital?
**Question 2**	What structures are in place in your hospital concerning in-hospital emergency medicine?
**Queries**	a) If an early warning system is in place: which early warning system for in-hospital emergencies do you use and when was it introduced?<br>b) If no early warning system is in place: do you have plans to implement an early warning system?
**Question 3**	What criteria do you use to raise the alarm in the event of an in-hospital emergency?
**Question 4**	Do you carry out quality control for in-hospital emergencies?
**Queries**	a) Are there any problems and/or obstacles that could lead to delayed emergency calls?
**Question 5**	What problems exist (could exist) when using the internationally established early warning system “MEWS” in the Austrian health system
**Question 6**	Are you aware of any other early warning systems for in-hospital emergencies than those already discussed?
**Question 7**	From your point of view and the point of view of the alerting staff/emergency teams, what does it need, so that early warning systems for in-hospital emergencies be accepted?
**Question 8**	What role does the management play in in-hospital emergency medicine?
**Question 9**	Has the SARS-CoV-2 pandemic led to a change in in-hospital emergencies?
**Closing**	a) Further information about data analysis

Sociodemographic data	
**Variable**	**Characteristics**
Gender	Male/female/diverse
Age	• 20 – 30 years<br>• 31 – 40 years<br>• 41 – 50 years<br>• 51 – 60 years<br>• Older than 60 years
Profession	Nurse/Physician/Others
Work experience	• Up to 3 years<br>• Between 4 and 7 years<br>• Between 8 and 15 years<br>• More than 16 years
Experience in in-hospital emergency medicine	• Between 3 and 5 years<br>• Between 6 and 8 years<br>• Between 9 and 11 years<br>• More than 12 years

Table S2. Annual rates of admissions, in-hospital emergencies and survival at discharge at Medical University Innsbruck

**Year**	**Admissions** *(n = 439701)*	**In-hospital emergencies** *(n = 1317)*	**IHCA** *(n = 365)*	**ROSC on scene** [a] *(n = 196)*	**24h survival of IHCA** *(n = 114)*
2017	80938	222	69 (31%) [b]	40 (58%) [c]	22 (32%) [c]
2018	81171	193	51 (26%)	25 (49%)	14 (27%)
2019	74615	216	58 (26%)	28 (48%)	18 (31%)
2020	65499	216	63 (29%)	35 (55%)	19 (30%)
2021	68898	212	65 (31%)	35 (54%)	20 (31%)
2022	68580	258	59 (23%)	33 (56%)	21 (36%)
Median	71724 (68660 – 74550)	216 (213 – 221)	61 (58 – 65)	34 (29 – 35)	20 (18 – 21)

Data presented as median (interquartile range) or number of patients. [a] Number of patients with ROSC after the end of the emergency dispatch. [b] % of in-hospital emergencies. [c] % of IHCA. Abbreviation: IHCA, in-hospital cardiac arrest. ROSC, return of spontaneous circulation.

Table S3. Code guideline

Category	Code Memo	Frequency of codes
Alerting Personnel	Statements that relate to alerting in in-hospital emergencies from the perspective of those raising the alert	7
Alerting Criteria	Statements that relate to criteria for alerting in in-hospital emergencies	17
Early Warning System	Statements that relate to early warning systems for the detection of in-hospital emergencies	47
Mode of Alerting	Statements that relate to the mode of alerting in in-hospital emergencies	11
Uniform Emergency Number	Statements relating to a uniform emergency number for alerting the in-hospital emergency team	15
Emergency Team – Structure	Statements relating to the structure of the emergency team in in-hospital emergency medicine	25
Emergency Team – Availability	Statements relating to the availability of the emergency team in in-hospital emergency medicine	8
Emergency Team – Competence	Statements relating to the competence of the emergency team in in-hospital emergency medicine	11
Administrative – Training	Statements relating to training and its organization in in-hospital emergency medicine	32
Administrative – Reporting	Statements that relate to reporting systems in in-hospital emergency medicine	14
Quality control	Statements relating to quality control in in-hospital emergency medicine	20
Financing	Statements that relate to the financing of in-hospital emergency medicine	12
Barriers and Obstacles	Statements relating to possible problems and obstacles in in-hospital emergency medicine	51
Role of Hospital Management	Statements relating to the role of hospital management in in-hospital emergency medicine	21
Retrospective view on the pandemic	Statements relating to changes due to the SARS-CoV-2 pandemic	25
Outlook	Statements relating to future developments	1
Requirements for EWS – the view of those alerting	Statements relating to prerequisites for the functioning of EWS from the perspective of the alerting personnel	26

Requirements for EWS – the view of those responding	Statements relating to prerequisites for the functioning of EWS from the perspective of the responding personnel	14
Resource shortage	Statements relating to an actual or potential shortage of resources	25
Future action	Statements relating to planned or future actions	41
Indentation reflects sub-codes (*n* = *9*). Abbreviation: EWS, early warning system.		423

Table S4. The five most frequent coded categories

**Code**	**Frequency**
Barriers and Obstacles	51 (12.1%)
Early Warning System	47 (11.1%)
Future action	41 (9.7%)
Administrative - Training	32 (7.6%)
Requirements for EWS – the view of those altering	26 (6.1%)

Abbreviation: EWS, early warning system.
